# HLA-DR/DQ eplet mismatch predicts *de novo* donor-specific antibody development in multi-ethnic Southeast Asian kidney transplant recipients on different immunosuppression regimens

**DOI:** 10.3389/fgene.2024.1447141

**Published:** 2024-08-28

**Authors:** Emmett Tsz Yeung Wong, Denise Pochinco, Anantharaman Vathsala, Wee Kun Koh, Amy Lim, Hersharan Kaur Sran, Matthew Ross D’Costa, Zi Yun Chang, Peter W. Nickerson, Chris Wiebe

**Affiliations:** ^1^ Department of Medicine, Yong Loo Lin School of Medicine, National University of Singapore, Singapore, Singapore; ^2^ National University Centre for Organ Transplantation, National University Hospital, Singapore, Singapore; ^3^ Department of Medicine, University of Manitoba, Winnipeg, MB, Canada; ^4^ Shared Health Services Manitoba, Winnipeg, MB, Canada; ^5^ Department of Immunology, University of Manitoba, Winnipeg, MB, Canada

**Keywords:** human leukocyte antigen, histocompatibility, molecular mismatch, eplets, kidney transplant, donor-specific antibodies

## Abstract

Eplet mismatch has been recognized as a more precise strategy for determining HLA compatibility by analyzing donor-recipient HLA differences at the molecular level. However, predicting post-transplant alloimmunity using single-molecule eplet mismatch categories has not been validated in Asian cohorts. We examined a cohort of Southeast Asian kidney transplant recipients (n = 234) to evaluate HLA-DR/DQ eplet mismatch as a predictor of *de novo* donor-specific antibody (dnDSA) development. HLA-DR/DQ single-molecule eplet mismatch was quantified using HLA Matchmaker, and we utilized previously published HLA-DR/DQ eplet mismatch thresholds to categorize recipients into alloimmune risk groups and evaluate their association with dnDSA development. Recognizing that the predominance of cyclosporine use (71%) may alter published eplet mismatch thresholds derived from a largely tacrolimus-based (87%) cohort, we evaluated cohort-specific thresholds for HLA-DR/DQ single-molecule eplet mismatch categories. Recipient ethnicities included Chinese (65%), Malays (17%), Indians (14%), and others (4%). HLA-DR/DQ dnDSA developed in 29/234 (12%) recipients after a median follow-up of 5.4 years, including against isolated HLA-DR (n = 7), isolated HLA-DQ (n = 11), or both (n = 11). HLA-DR/DQ single-molecule eplet mismatch risk categories correlated with dnDSA-free survival (*p* = 0.001) with low-risk recipients having a dnDSA prevalence of 1% over 5 years. The cohort-specific alloimmune risk categories improved correlation with HLA-DR/DQ dnDSA-free survival and remained significant after adjusting for calcineurin inhibitor and anti-metabolite immunosuppression (*p* < 0.001). We validated the performance of single-molecule eplet mismatch categories as a prognostic biomarker for HLA-DR/DQ dnDSA development in a cohort of predominantly Asian kidney transplant recipients after adjusting for different immunosuppression regimens.

## 1 Introduction

**FIGURE 1:**
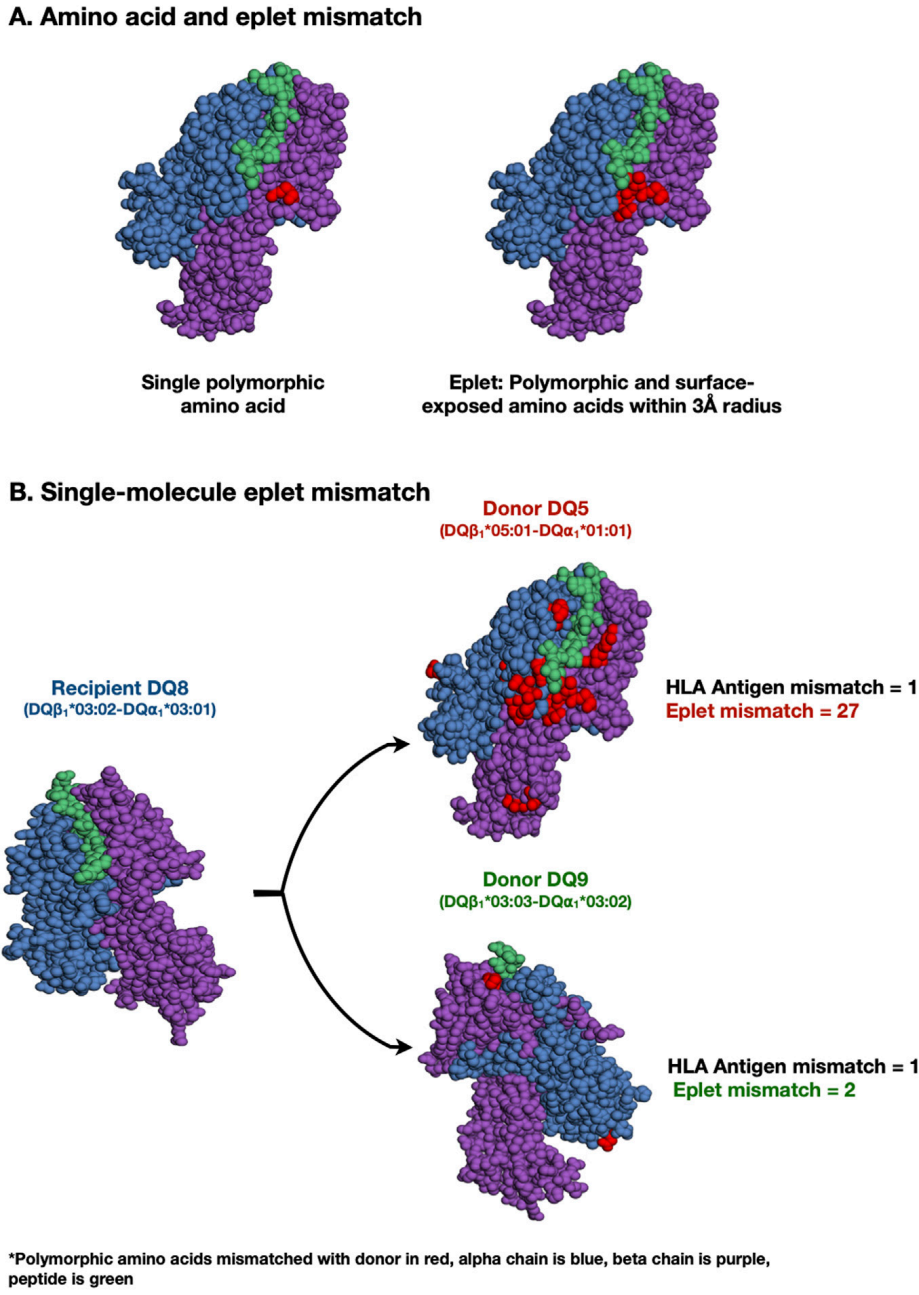
Amino acid, eplet, and single-molecule eplet mismatch. A mismatched polymorphic amino acid (red) is the most basic unit of HLA mismatch. Eplets are single or small patches of mismatched polymorphic amino acids within a 3-Å radius exposed near an HLA molecule’s surface. Eplet mismatch has been recognized as a more precise strategy for determining HLA compatibility by enhancing the quantification of donor-recipient HLA difference at the molecular level (1A). A theoretical example of single-molecule eplet mismatch at HLA-DQ locus. The eplet mismatch for each donor HLA-DQ⍺_1_β_1_ molecule is determined individually by comparing each molecule with all recipient HLA-DQ⍺_1_β_1_ molecules. The maximum single-molecule eplet mismatch for each recipient is the greatest HLA-DQα_1_β_1_ eplet mismatch, which was correlated with *de novo* DSA development at HLA-DQ. Two different HLA mismatches can have significantly different single-molecule eplet mismatches despite both having one HLA antigen mismatch. (1B).

Alloimmune-mediated injury is the most common cause of kidney transplant allograft failure (Bouatou et al., 2019; El-Zoghby et al., 2009; Sellarés et al., 2012). In the absence of pre-formed HLA donor-specific antibody (i.e., memory), *de novo* donor-specific antibody (dnDSA) development has been established as a biomarker of post-transplant primary alloimmunity and is associated with rejection and reduced allograft survival (Wiebe et al., 2012; Wiebe et al., 2017; Wiebe et al., 2019; Senev et al., 2020a). Traditionally, HLA compatibility between donor and recipient has been determined at the HLA-A, -B, and -DR antigen levels. Over the past decade, eplet mismatch (patches of polymorphic surface-exposed amino acids within a 3-Å radius, Figure 1A) has been recognized as a more precise strategy for determining HLA compatibility by enhancing the quantification of donor-recipient HLA difference at the molecular level (Duquesnoy and Askar, 2007).

HLA Matchmaker, a structurally based computer algorithm, is the most frequently used software to determine the eplet mismatch load between donor and recipient (Duquesnoy, 2006). In brief, it compares amino acid sequences between donor and recipient alleles, incorporates the three-dimensional location of these amino acids, and identifies surface-exposed patches that are brought into proximity on the tertiary structure that may constitute the binding sites for complementarity-determining regions of anti-HLA antibody paratopes. HLA Class II eplet mismatch determination has been shown to be superior to HLA antigen mismatch for predicting Class II dnDSA development, T cell-mediated rejection, and antibody-mediated rejection (Wiebe et al., 2019; Wiebe et al., 2013; Wiebe et al., 2015; Kosmoliaptsis et al., 2016).

Asian ethnicities have been underrepresented in studies validating the application of single-molecule eplet mismatch in predicting post-transplant alloimmune outcomes. In this report, we examined a cohort of multi-ethnic Southeast Asian kidney transplant recipients to validate the use of eplet mismatch and alloimmune risk categories as predictors of dnDSA development.

## 2 Methods

This single-center cohort consisted of 319 consecutive adult kidney transplant recipients at the National University Centre for Organ Transplantation (NUCOT), National University Hospital, Singapore, between January 2013 and December 2022, with at least 6 months of follow-up at our center. Approval was obtained from the NHG Domain Specific Review Board under various protocols (2014/01024, 2018/00900, 2015/00905, 2021/01164, 2021/00630), and the study was conducted in accordance with the Declaration of Helsinki.

Donor and recipient demographics and clinical information were extracted from electronic medical records. Recipients were excluded for primary non-function (n = 1), simultaneous pancreas-kidney transplants (n = 3), and blood-group ABO-incompatible transplants (n = 16). We also excluded recipients with proven or possible memory alloimmunity, including recipients with pre-transplant DSA (n = 33), crossmatch positivity (n = 16), inadequate evaluation for pre-transplant DSA (n = 6), and early post-transplant DSA within 14 days (n = 10), leaving 234 for analysis.

### 2.1 HLA typing and eplet mismatch identification

Recipient and donor HLA typing at HLA-A, -B, -C, -DRB1, and -DRB345 were determined serologically with complement-dependent cytotoxicity (CDC) methods (up to September 2009) or at low-resolution (one-field) with DNA-based sequence-specific primer (PCR-SSP) technology (LABType, Micro SSP™; One Lambda, Canoga Park, CA or Morgan™ HLA SSP; Texas BioGene Inc., Richardson, TX). Where clinically indicated, typing at intermediate-to high-resolution (two-field) for particular loci of interest was performed by PCR-SSP or sequence-based testing (SBT, SeCore™; One Lambda, Canoga Park, CA). HLA typing at HLA-DQB1 was performed with low-resolution PCR-SSP (up to 2019) or at intermediate-to high-resolution with PCR-SSP or SBT, and HLA-DQA1, where available, was typed at intermediate-to high-resolution with PCR-SSP or SBT. Select recipients and donors had HLA typing performed at high resolution using next-generation sequencing (NGS, AllType™; One Lambda, Canoga Park, CA) when the method was made available in the HLA laboratory after July 2023. The proportion of recipients whose HLA-DR and -DQ loci typing were performed with each method is shown in Supplementary Table S1.

For recipients and donors with only low-resolution typing, or where 2-field typing was not available at HLA-DR and HLA-DQ loci, 4-digit HLA alleles were imputed using knowledge of HLA allele associations in the individual’s closest ethnic group using the 2011 dataset available on the HaploStats application (https://www.haplostats.org), a web application provided by the National Marrow Donor Program (NMDP) Bioinformatics group.

HLA Matchmaker software (HLA DRDQDP Eplet Matching version 2.2, http://www.hlamatchmaker.net) was used to determine the eplet mismatch for each molecule individually at each HLA-DR and -DQ locus and the maximum single-molecule mismatch for each recipient (greatest HLA-DRβ_1/3/4/5_ or HLA-DQα_1_β_1_ eplet mismatch after comparing each donor allele with all recipient alleles, illustrative example in Figure 1B) was correlated with dnDSA development at that locus.

The single-molecule eplet mismatch threshold for HLA-DR was determined by receiver operating characteristic (ROC) curve analysis of individual HLA-DR molecules correlated with dnDSA development against these HLA-DR molecules, while the eplet mismatch threshold for HLA-DQ was similarly determined by ROC curve analysis of individual HLA-DQ molecules correlated with dnDSA development against these HLA-DQ molecules. For HLA-DR, each maternal and paternal HLA-DRβ_1_ (n = 468), HLA-DRβ_3_ (n = 192), HLA-DRβ_4_ (n = 127), HLA-DRβ_5_ (n = 104) donor alleles were considered. Donor null alleles at HLA-DRβ_3/4/5_ (n = 45) did not count toward the total. For HLA-DQ, alpha and beta alleles inherited as a haplotype were considered as one HLA-DQα_1_β_1_ molecule (n = 468). The most common allele HLA-DR and HLA-DQ allele frequencies at each locus for donors and recipients of the three main ethnicities (Chinese, Malay, and Indian) can be found in Supplementary Table S2.

### 2.2 Antibody assessment and monitoring

All included recipients in the analysis had remote or immediate pre-transplant sera screened by panel reactive antibody assays (CDC PRA up to early 2018, FlowPRA™; One Lambda, Canoga Park, CA after) and/or single antigen beads (SAB, LABScreen™; One Lambda, Canoga Park, CA). Pre-transplant antibody assessment was considered adequate if SAB testing was performed or PRA was zero by the CDC or FlowPRA, in conjunction with a negative crossmatch. Crossmatch positivity was defined as positive if CDC crossmatch was positive after treatment with anti-human globulin (AHG) or AHG/Dithiothreitol (DTT) or if flow crossmatch was positive after pronase treatment.

DSA screening was performed at least once a year post-transplant or at the time of biopsy for allograft dysfunction. Recipients were screened with FlowPRA™ beads representing HLA-A, -B, -Cw, -DR, -DQ, and -DP antigens. If the screening assay was positive, HLA antibody specificities were validated using LABScreen™ SAB using a threshold mean fluorescence intensity (MFI) value of ≥500. We defined any DSA detected in the first 14 days post-transplant as a memory response.

### 2.3 Statistical analysis

Means and standard deviations (SD), median and interquartile ranges (IQR), or counts and percentages were used to describe the baseline characteristics of the cohort. Comparisons between baseline predictors and clinical outcomes were performed using the Wilcoxon-rank test for non-parametric data. Comparisons across groups were performed using the Kruskal–Wallis test for non-parametric variables. Survival analyses were performed by the Kaplan-Meier method using the log-rank test for significance. Associations of baseline covariates and dnDSA-free survival were performed using conventional Cox proportional hazards models. Analyses were conducted using JMP Pro (version 16.2).

## 3 Results

### 3.1 Study population

This cohort had a median follow-up of 5.4 years (IQR 3.3–8.0). The median 5- and 10-year all-cause allograft survival were 90% and 81%, and the 5- and 10-year death-censored allograft survival were 97% and 90% respectively. Baseline recipient demographics can be found in Table 1. This cohort was predominantly Chinese (65%) but also included Malays (17%), Indian (14%), or other Southeast Asian ethnicities (Vietnamese, Myanmarese, Bruneian, Filipino); 44% received kidneys from deceased donors. Among recipients who received a kidney from living donors, 57% were from a biologically related donor. Standard immunosuppression consisted of prednisolone in all recipients, a calcineurin inhibitor (cyclosporine 71%; tacrolimus 29%), and an anti-metabolite (mycophenolate mofetil/mycophenolic acid 88%; azathioprine 11%). Induction therapy was used for all recipients with thymoglobulin (19%) or basiliximab (81%).

**TABLE 1:** Baseline recipient demographics (n = 234).

Male Donor	41%
Donor Age (years)	49 ± 14
Deceased Donor	44%
Previous Transplant	5%
Pre-emptive Transplant	11%
Male Recipient	54%
Recipient Age (years)	47 ± 12
Recipient Ethnicity	
Chinese	65%
Malay	17%
Indian	14%
Time from Dialysis to Transplant (years)	4.1 (0.8, 8.2)
Delayed Graft Function	15%
Induction Immunosuppression	
Basiliximab	81%
Thymoglobulin	19%
Calcineurin Inhibitor	
Cyclosporine	71%
Tacrolimus	29%
Anti-metabolite	
Mycophenolate	88%
Azathioprine	11%
None	1%
Single-molecule eplet mismatch alloimmune category (Wiebe et al.) (Wiebe et al., 2019)	
Low (HLA-DR <7 and HLA-DQ <9)	30%
Intermediate (Not meeting the low-risk definition and HLA-DQ <15)	35%
High (HLA-DQ ≥15)	35%

*De novo* DSA developed in 39 out of 234 (17%) recipients, including 7/39 (18%) to HLA Class I, 20/39 (51%) to Class II, and 12/39 (31%) to both Class I and Class II antigens, respectively. Recipients who developed Class II dnDSA with or without Class I had decreased death-censored allograft survival (HR 6.2, 95% CI 1.5–25, *p* = 0.01) compared to recipients who did not develop dnDSA (Supplementary Figure S1). No recipients with isolated Class I dnDSA (n = 7) or isolated HLA-DP dnDSA (n = 2) experienced allograft loss.

### 3.2 HLA-DR/DQ single-molecule eplet mismatch correlated with HLA-DR/DQ dnDSA development

HLA-DR/DQ dnDSA developed in 29/234 (12%) recipients (range 2–89 months post-transplant), including against isolated HLA-DR (n = 7), isolated HLA-DQ (n = 11), or both (n = 11). HLA‐DRβ_1/3/4/5_ single-molecule eplet mismatches (range 0–21) correlated with HLA-DR dnDSA development (area under the curve, AUC 0.88). HLA‐DQα_1_β_1_ single-molecule eplet mismatches (range 0–32) correlated with HLA-DQ dnDSA development (AUC 0.78).

The maximum single-molecule eplet mismatch (greatest HLA-DRβ_1/3/4/5_ or HLA-DQα_1_β_1_ eplet mismatch after comparing each donor allele with all recipient alleles) correlated with dnDSA-free survival for both HLA-DR (HR 1.3 per eplet mismatch, 95% CI 1.2–1.4, *p* < 0.0001) and HLA-DQ (HR 1.1, 95% CI 1.0–1.1, *p* < 0.01). In a sensitivity analysis excluding recipients with zero eplet mismatch at each locus, this correlation remained for both HLA-DR (HR 1.3, 95% CI 1.2–1.4, *p* < 0.0001) and HLA-DQ (HR 1.1, 95% CI 1.0–1.1, *p* = 0.047).

### 3.3 HLA-DR/DQ single-molecule eplet mismatch categories

**FIGURE 2:**
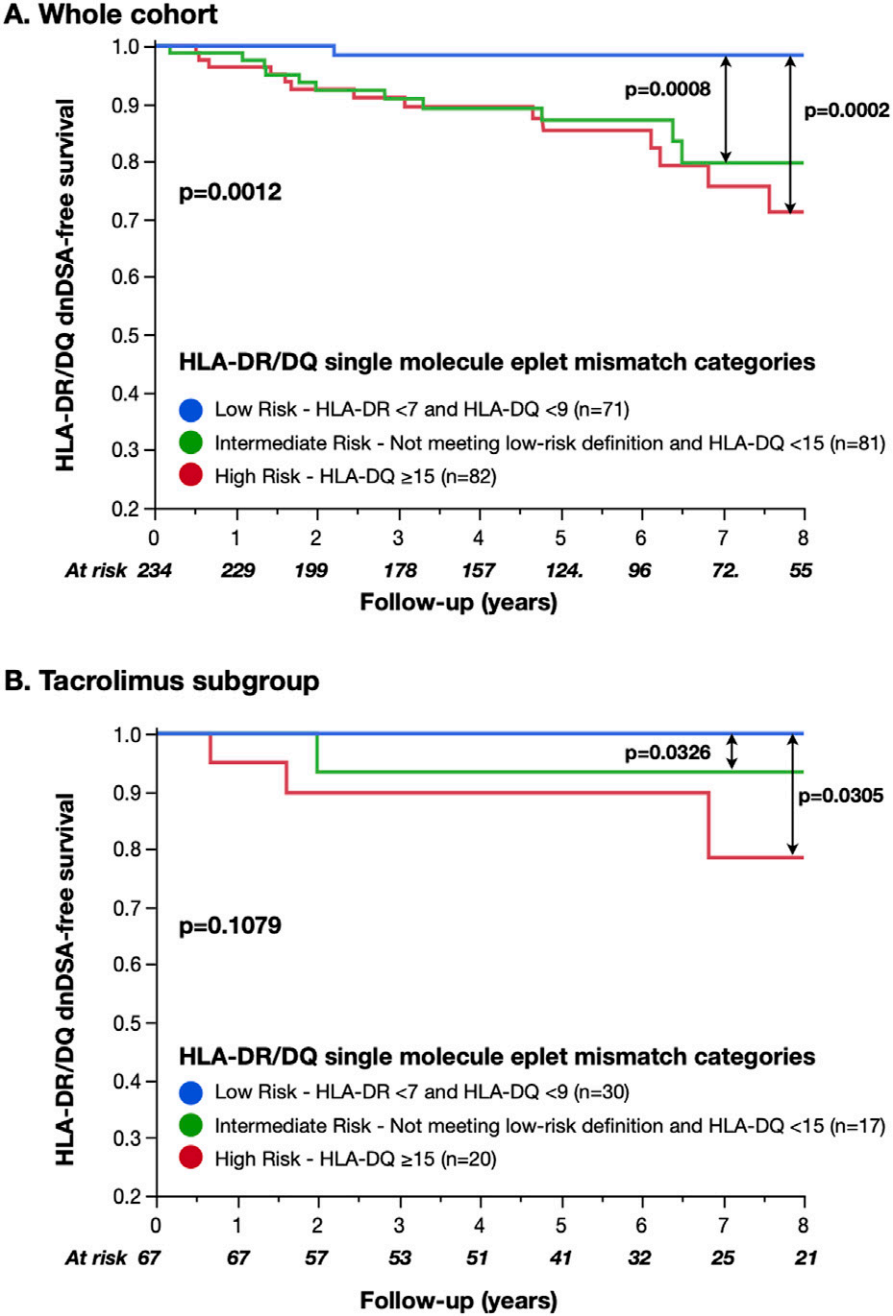
HLA-DR/DQ dnDSA-free survival by Wiebe et al. single-molecule eplet mismatch categories. Intermediate and high-risk HLA-DR/DQ single-molecule eplet mismatch alloimmune categories significantly correlated with reduced HLA-DR/DQ dnDSA-free survival compared to the low-risk category. However, no difference was observed between the intermediate- and high-risk categories (2A). Although non-significant, the correlation between alloimmune risk categories and dnDSA prevalence in the tacrolimus subset showed a layering of effect similar to that of previously published cohorts (Wiebe et al., 2019; Johnson et al., 2023). The small number of recipients and dnDSA events limited statistical comparisons within the subset (2B).

We categorized recipients into previously published HLA-DR/DQ alloimmune risk groups (Low-risk, maximum HLA-DR eplet mismatch <7 and HLA-DQ <9; Intermediate-risk, not meeting the low-risk definition and HLA-DQ <15; and High-risk, HLA-DQ ≥15) (Wiebe et al., 2019). The proportion of recipients classified as low, intermediate, and high alloimmune risk was 30%, 35%, and 35% respectively (Table 2). In recipients categorized as low-risk by HLA-DR/DQ single-molecule eplet mismatch, only 1/71 (1%) developed HLA-DR/DQ dnDSA. HLA-DR/DQ single-molecule eplet mismatch alloimmune categories correlated with HLA-DR/DQ dnDSA-free survival (low vs intermediate *p* < 0.001, intermediate vs high *p* = 0.7, and low vs high *p* < 0.001, Figure 2A).

**TABLE 2:** Characteristics of recipients in cohorts that have applied HLA-DR/DQ single-molecule eplet mismatch alloimmune categories for HLA-DR/DQ dnDSA prediction.

	Manitoba (Wiebe et al.) (Wiebe et al., 2019)	Denver (Davis et al.) (Davis et al., 2021)	Leuven (Senev et al.) (Senev et al., 2020a)	Emory (Johnson et al.) (Johnson et al., 2023)	NUCOT, Singapore
Predominant ethnicity	Caucasian (67%)	Caucasian (72%)	Caucasian (98%)	African-American (56%)	Asian (100%)
Second most common ethnicity	Indigenous (19%)	Hispanic (16%)	N/A	Caucasian (34%)	N/A
Single-molecule eplet mismatch categories					
Low	25%	27%	40%	21%	30%
Intermediate	35%	34%	32%	33%	35%
High	40%	39%	28%	46%	35%
Induction immunosuppression					
Basiliximab	18%	3%*	36%	100%	81%
Thymoglobulin	21%	42%	2%	0%	19%
None	16%	55%	59%	0%	0%
Maintenance immunosuppression					
Mycophenolate, tacrolimus and corticosteroids	87%	93%	87%	100%^†^	16%
HLA-DR/DQ dnDSA development rate in low-risk recipients	1%	2%	1%	0%	1%
Median follow-up	7.5 years	1 year	7.5 years	3 years	5.4 years

*Includes other induction therapies.

^†^Fifty percent of recipients were on belatacept with transient co-immunosuppression with tacrolimus, which ceased at 1 year.

Since the published alloimmune risk thresholds were developed in a cohort receiving predominantly tacrolimus-based immunosuppression (87% *versus* 29% in the current cohort), we performed a subgroup analysis limited to recipients treated with tacrolimus (n = 67). In this subset, there was a trend toward alloimmune risk categories correlating with dnDSA-free survival (*p* = 0.1, Figure 2B); however, the small number of recipients and events limited statistical comparisons.

### 3.4 Defining NUCOT cohort-specific alloimmune risk categories

Recognizing that the predominance of cyclosporine use in the study cohort may alter eplet mismatch thresholds for dnDSA development, we evaluated cohort-specific thresholds for HLA-DR/DQ single-molecule eplet mismatches to define alloimmune risk categories (referred to as NUCOT alloimmune risk categories below). Similar to previously published work, we also found that recipients with maximum HLA‐DRβ_1/3/4/5_ eplet mismatch <7 and HLA-DQα_1_β_1_ < 9 had a low prevalence of dnDSA development (1% after 5 years of follow-up). Therefore, these thresholds were retained as the NUCOT low-risk category. To define the intermediate and high-risk categories, we re-examined the correlation between dnDSA development and eplet mismatch by ROC analysis after excluding the recipients (n = 71) already defined as low-risk. This analysis identified an HLA-DR single-molecule eplet mismatch threshold of 12 and an HLA-DQ single-molecule eplet mismatch threshold of 15, respectively. We thus defined the NUCOT high-risk category as HLA-DR ≥12 and HLA-DQ ≥15. Recipients not meeting the low- or high-risk criteria were categorized as intermediate-risk. The proportion of recipients classified as NUCOT low, intermediate, and high alloimmune risk was 30%, 55%, and 15%, respectively.

**FIGURE 3:**
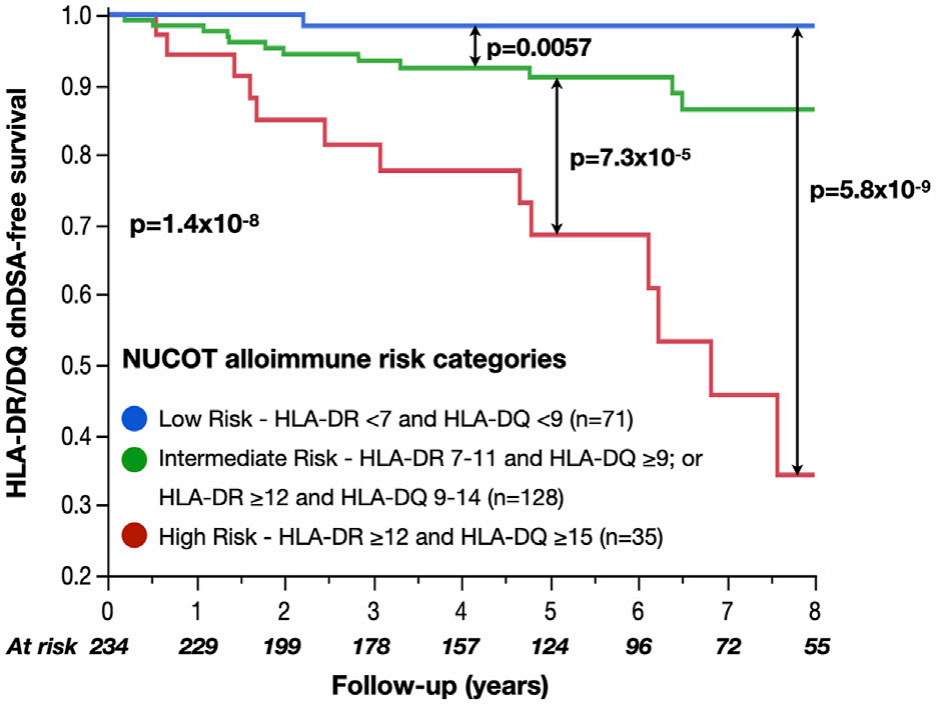
HLA-DR/DQ dnDSA-free survival by NUCOT alloimmune risk categories NUCOT alloimmune risk categories significantly correlated with HLA-DR/DQ dnDSA-free survival.

NUCOT alloimmune risk categories significantly correlated with HLA-DR/DQ dnDSA-free survival (low vs intermediate *p* < 0.01, intermediate vs high *p* < 0.0001, and low vs high *p* < 0.0001, Figure 3). Immunosuppression (calcineurin inhibitors, anti-metabolite) and NUCOT alloimmune risk categories were included in the multivariate model due to historical correlation with dnDSA development in the literature and *p* < 0.2 in univariate analyses (Supplementary Table S3). After adjusting for calcineurin inhibitor (cyclosporine vs tacrolimus HR 1.7, 95% CI 0.6–4.8, *p* = 0.4) and anti-metabolite immunosuppression (mycophenolate vs azathioprine HR 1.6, 95% CI 0.2–14.5, *p* = 0.7) prescribed on discharge, only NUCOT alloimmune risk categories correlated with HLA-DR/DQ dnDSA-free survival (intermediate vs low HR 8.8, 95% CI 1.1–67.0, *p* = 0.04, high vs intermediate HR 4.1, 95% CI 1.9–8.8, *p* < 0.001, and high vs low HR 36.2, 95% CI 4.7–282, *p* < 0.001, Table 3).

**TABLE 3:** NUCOT HLA-DR/DQ alloimmune risk categories correlated with dnDSA development after adjustment for maintenance immunosuppression.

	Adjusted Hazard Ratio (95% CI)	*p*-value
Calcineurin Inhibitor (Cyclosporine vs Tacrolimus)	1.66 (0.57, 4.81)	0.3525
Anti-metabolite (Mycophenolate vs Azathioprine)	1.60 (0.18, 14.5)	0.6771
NUCOT Alloimmune Risk Categories		
Intermediate vs Low	8.76 (1.15, 67.0)	**0.0365**
High vs Intermediate	4.13 (1.95, 8.77)	**0.0002**
High vs Low	36.2 (4.65, 282)	**0.0006**

## 4 Discussion

This study validated the application of single-molecule eplet mismatch categorization to predict the risk of HLA-DR/DQ dnDSA development in a multi-ethnic Southeast Asian cohort. We found that recipients categorized as low-risk had an HLA-DR/DQ dnDSA prevalence of 1% after 5 years of routine DSA surveillance. However, no difference in HLA-DR/DQ dnDSA-free survival was found between the intermediate and high alloimmune risk categories. Recognizing that the predominance of cyclosporine use may alter published eplet mismatch thresholds derived from a largely tacrolimus-based (87%) cohort, we evaluated NUCOT cohort-specific thresholds for HLA-DR/DQ single-molecule eplet mismatch categories. The NUCOT alloimmune risk categories improved correlation with HLA-DR/DQ dnDSA-free survival, suggesting that alternative thresholds may need to be considered for cohorts where cyclosporine is the predominant calcineurin inhibitor choice.

Previous studies using the HLA-DR/DQ single-molecule eplet categories included predominantly Caucasian (i.e., Wiebe et al. 67%, Davis et al. 72%, Senev et al. 98%) or African-American (Johnson et al. 56%) ethnicities (Table 2). (Wiebe et al., 2019; Senev et al., 2020a; Davis et al., 2021; Johnson et al., 2023) Furthermore, Indigenous 19% and Hispanic 16% populations were well represented in the Wiebe et al. and Davis et al. studies, respectively (Wiebe et al., 2019; Davis et al., 2021). The proportion of low-risk recipients (maximum HLA-DR <7 and HLA-DQ <9) in these cohorts varied from 21%–40% with a dnDSA prevalence of 0%–2% over a median follow-up range of 1–7.5 years. In the current study, the first with predominantly Asian ethnicity, 30% were low risk with a 1% prevalence of dnDSA at 5.4 years. Notably, these low rates of dnDSA development were consistently observed despite variations in racial composition, induction, and maintenance immunosuppression (Table 2). This strengthens the evidence that low-risk alloimmune categories correlate with a low prevalence of dnDSA development across geographic boundaries, ethnic differences, and immunosuppression selection.

Since each antigen mismatch results in a wide range of eplet mismatches for HLA-DRβ_1/3/4/5_ (0–21) and HLA-DQα_1_β_1_ (0–32), antigen mismatch is not a dependable correlate of primary alloimmune risk. Thus, antigen mismatch can only reliably predict low risk for primary alloimmunity in recipients with zero mismatches. Single-molecule eplet mismatch provides a more precise and granular method of assessing the HLA-DR/DQ dnDSA development risk. Whereas n = 41 recipients had zero HLA-DRβ_1_/DQβ_1_ antigen mismatch, n = 71 recipients were categorized as NUCOT low-risk for HLA-DR/DQ dnDSA development. This represented a 73% increase in the number of recipients categorized as low risk despite similar rates of dnDSA development in each group (n = 1/41, 2% vs. n = 1/71, 1%). Even after excluding recipients with zero HLA-DR/DQ eplet mismatch (n = 24/234, 10%), NUCOT low-risk recipients had a low rate of dnDSA development (n = 1/47, 2% at 5 years). Potential applications include immunosuppression and dnDSA surveillance strategies tailored to the risk of primary alloimmunity.

While HLA-DR/DQ single-molecule eplet mismatch has previously been studied in cohorts predominantly treated with tacrolimus-based triple immunosuppression (i.e., Wiebe et al. 87%, Davis et al. 100%, Senev et al. 87%, and Johnson et al. 100%, Table 2) (Wiebe et al., 2019; Senev et al., 2020a; Davis et al., 2021; Johnson et al., 2023), the current study differs with 71% cyclosporine use. We observed no difference in the HLA-DR/DQ dnDSA-free survival between the intermediate- and high-risk groups using previously published thresholds for alloimmune risk categorization, although there was a trend when the subset treated with tacrolimus were analyzed (n = 67, n = 5 events, Figure 2B). (Wiebe et al., 2019) Postulating that the predominance of cyclosporine use in the study cohort may alter the eplet mismatch thresholds for dnDSA development, we evaluated the HLA-DR/DQ single-molecule eplet mismatch cut-offs to define cohort-specific NUCOT intermediate- and high-risk alloimmune categories. We found identical thresholds to define low-risk and similar HLA-DQ eplet mismatch thresholds (≥15) to define high-risk. However, in the NUCOT cohort, an additional HLA-DR eplet mismatch threshold of ≥12 improved risk prediction, unlike the Wiebe et al. cohort, where intermediate- and high-risk groups were determined by HLA-DQ eplet mismatch alone. Further study will be required to investigate the relative importance of HLA-DR eplet mismatch on HLA-DR dnDSA development in cohorts using cyclosporine.

In the current study, cyclosporine was associated with an increased risk of dnDSA development (HR 1.7, *p* = 0.4), consistent with that reported in the larger Wiebe et al.’s study (HR 2.1, *p* < 0.01) (Wiebe et al., 2019). The Kidney Disease: Improving Global Outcomes (KDIGO) Clinical Practice Guidelines 2009 have suggested that tacrolimus should be the first-line calcineurin inhibitor for kidney transplant recipients (Level of recommendation 2A) following several trials comparing cyclosporine with tacrolimus (Eckardt et al., 2009). Several cohorts and registries reported that the proportion of kidney transplant recipients who receive tacrolimus-based immunosuppression regimens ranges from 70 to >90% (Axelrod et al., 2016; Chang et al., 2017; Rosa et al., 2023; OPTN/SRTR, 2022 Annual Data Report, 2024). However, due to the high incidence of end-stage kidney disease attributable to diabetes mellitus in Singapore (2019 USRDS Annual Data Report, 2019), increased susceptibility of South Asians to post-transplant diabetes mellitus (Peracha et al., 2016), and the association of tacrolimus with post-transplant diabetes (Webster et al., 2005), our center has historically used cyclosporine as the most common choice of calcineurin inhibitor. Nevertheless, NUCOT low-risk alloimmune category recipients had a low absolute risk of dnDSA development (n = 1/71, 1%) despite the increased relative risk corresponding to immunosuppression choice. On the contrary, NUCOT high-risk recipients had a significantly increased risk of HLA-DR/DQ dnDSA development compared to low-risk recipients (HR 36.2, *p* = 0.0006) regardless of immunosuppressive therapy. Although the magnitude of risk and wide confidence interval needs to be interpreted in the context of small cohort size, Davis et al. reported similar hazard ratios (HR 21.6, *p* < 0.001) between their low-risk and high-risk groups in their larger cohort (n = 444) in which all recipients were treated with tacrolimus (Davis et al., 2021). This underscores that recipients categorized as high-risk by single-molecule eplet mismatch represent a subgroup most likely to benefit from increased monitoring to capture medication adherence, avoidance of immunosuppression minimization, and tacrolimus-based immunosuppression.

There is currently no consensus regarding routine surveillance for dnDSA beyond 18 months post-transplant due to the lack of treatment options and resources required (Tait et al., 2013; Archdeacon et al., 2011). This study provides supportive evidence for a novel data-driven, risk-based approach to selecting recipients most likely to benefit from dnDSA surveillance recently published (Wiebe et al., 2023). In this context, recipients at low risk for dnDSA development could have their dnDSA surveillance minimized or discontinued, offsetting or exceeding any potential increase in resources required to perform molecule mismatch assessment. These recipients may also be potential candidates for consideration of physician-directed immunosuppression minimization; however, prospective trials are required to test this hypothesis prior to implementation.

## 5 Limitations

Due to the relatively small cohort size and subgroups with different immunosuppression regimens and ethnicities, we cannot exclude the risk of type II error. Risk quantification should be interpreted with caution and validated in independent cohorts. Cohorts with different baseline demographics, pre-transplant DSA screening protocols, or post-transplant immunosuppression protocols may experience different rates of dnDSA development.

As PRA assays are less sensitive than SAB assays as screening tools, we were unable to exclude the possibility of missed weak pre-transplant DSAs, which did not result in a positive crossmatch.

Several groups have reported that when low-resolution two-digit typing is used to impute high-resolution typing, there are discrepancies compared to next-generation sequencing (NGS), particularly within ethnic minorities or ethnicities not well-represented in these databases or registries (Senev et al., 2020b; Engen et al., 2020; D’Souza et al., 2018). Nevertheless, >90% of imputed results in these studies were within +/- 3 eplet mismatches of the NGS results, suggesting most recipients would remain in the same alloimmune risk categories. This is supported by a recent study by Cohen et al. that showed that imputation from low-resolution typing resulted in little impact on alloimmune risk categorization in racially concordant and discordant donor-recipient pairs (Cohen et al., 2024). Nevertheless, as HLA typing methodology improves, it is reasonable to expect further improvement in the correlation between molecular mismatch and alloimmune outcomes. Within these limitations, HLA-DR/DQ single-molecule eplet mismatch has been predictive of dnDSA in other cohorts with significantly different ethnic compositions (Wiebe et al., 2019; Senev et al., 2020a; Davis et al., 2021; Johnson et al., 2023). We did not examine associations between HLA-DR/DQ eplet mismatch and rejection outcomes. Immunosuppression drug levels were also not analyzed in this study, thus we are unable to exclude the impact of immunosuppression minimization, non-adherence, or subsequent changes in immunosuppression regimens on dnDSA development.

## 6 Conclusion

HLA-DR/DQ eplet mismatch correlated with dnDSA development in our multi-ethnic cohort of Southeast Asian kidney transplant recipients. We validated the performance of single-molecule eplet mismatch categories as a prognostic biomarker in stratifying recipients into low-, intermediate-, and high-risk for dnDSA development. HLA-DR/DQ eplet thresholds for categorizing recipients as low-risk appear reproducible despite geographic, ethnic, and immunosuppression differences, identifying a group that may benefit from reduced dnDSA surveillance. This provides further evidence for the role of molecular mismatch in precision medicine in kidney transplantation.

## Data Availability

The datasets presented in this article are not readily available because of privacy or ethical restrictions. Requests to access the datasets should be directed to EW, e.wong@nus.edu.sg.
