# Supplementary material for: HLA-DR/DQ eplet mismatch predicts de novo donor-specific antibody development in multi-ethnic Southeast Asian kidney transplant recipients on different immunosuppression regimens
Source: Front Genet. 2024 Aug 28;15:1447141. doi: 10.3389/fgene.2024.1447141 (PMC11387181; doi:10.3389/fgene.2024.1447141)
Supplement: Supplementary file 1 [file Table1.DOCX]

**Supplementary Material**

**Supplementary Table 1. The proportion of recipients whose HLA-DR and HLA-DQ typing were performed with each method**

|  | **HLA-DR** | **HLA-DQB1** | **HLA-DQA1** |
| --- | --- | --- | --- |
| Complement-dependent cytotoxicity | 9% | - | - |
| PCR-SSP (one-field)  One Lambda Micro SSP^TM^ Generic Trays /  Texas BioGene Morgan^TM^ HLA SSP Typing Kit | 82% | 61% | - |
| PCR-SSP / SBT (two-field)  One Lambda Micro SSP^TM^ High Resolution Trays /  One Lambda SeCore^TM^ | 9% | 39% | 100% |
| PCR-SSP: Polymerase Chain Reaction Sequence-Specific Primer; SBT: Sequence-Based Testing | | | |

**Supplementary Table 2. The most common donor and recipient HLA-DR and HLA-DQ allele frequencies**

|  | **Chinese**  **(n=312)** | **Malay**  **(n=69)** | **Indian**  **(n=61)** |
| --- | --- | --- | --- |
| **HLA-DRB1** | DRβ_1_*09:01 (12%)  DRβ_1_*12:02 (12%)  DRβ_1_*15:01 (10%)  DRβ_1_*08:03 (9%)  DRβ_1_*11:01 (9%) | DRβ_1_*12:02 (35%)  DRβ_1_*15:02 (19%)  DRβ_1_*07:01 (11%)  DRβ_1_*15:01 (6%)  DRβ_1_*16:02 (4%) | DRβ_1_*15:02 (13%)  DRβ_1_*07:01 (12%)  DRβ_1_*14:04 (11%)  DRβ_1_*03:01 (9%)  DRβ_1_*10:01 (9%) |
| **HLA-DRB3** | DRβ_3_*02:02 (64%)  DRβ_3_*03:01 (28%)  DRβ_3_*01:01 (8%) | DRβ_3_*03:01 (75%)  DRβ_3_*02:02 (19%)  DRβ_3_*01:01 (6%) | DRβ_3_*02:02 (67%)  DRβ_3_*03:01 (23%)  DRβ_3_*01:01 (10%) |
| **HLA-DRB4** | DRβ_4_*01:03 (84%)  DRβ_4_*01:01 (16%) | DRβ_4_*01:03 (95%)  DRβ_4_*01:01 (5%) | DRβ_4_*01:03 (82%)  DRβ_4_*01:01 (18%) |
| **HLA-DRB5** | DRβ_5_*01:01 (93%)  DRβ_5_*01:02 (6%)  DRβ_5_*02:02 (1%) | DRβ_5_*01:01 (62%)  DRβ_5_*01:02 (35%)  DRβ_5_*02:02 (4%) | DRβ_5_*01:01 (50%)  DRβ_5_*01:02 (46%)  DRβ_5_*02:02 (4%) |
| **HLA-DQB1** | DQβ_1_*03:01 (25%)  DQβ_1_*06:01 (14%)  DQβ_1_*03:03 (12%)  DQβ_1_*02:01 (8%)  DQβ_1_*03:02 (9%) | DQβ_1_*03:01 (36%)  DQβ_1_*05:01 (12%)  DQβ_1_*06:01 (12%)  DQβ_1_*05:02 (10%)  DQβ_1_*02:01 (7%) | DQβ_1_*06:01 (19%)  DQβ_1_*02:01 (13%)  DQβ_1_*03:01 (13%)  DQβ_1_*05:01 (13%)  DQβ_1_*05:03 (11%) |
| **HLA-DQA1** | DQ⍺_1_*01:02 (16%)  DQ⍺_1_*03:02 (12%)  DQ⍺_1_*06:01 (11%)  DQ⍺_1_*05:05 (11%)  DQ⍺_1_*01:03 (10%) | DQ⍺_1_*06:01 (34%)  DQ⍺_1_*01:02 (20%)  DQ⍺_1_*01:01 (13%)  DQ⍺_1_*02:01 (11%)  DQ⍺_1_*01:03 (7%) | DQ⍺_1_*01:03 (20%)  DQ⍺_1_*02:01 (13%)  DQ⍺_1_*01:02 (11%)  DQ⍺_1_*01:04 (11%)  DQ⍺_1_*01:05 (9%) |

**Supplementary Table 3. Baseline demographics as predictors of dnDSA development**

|  | **Hazard Ratio (95% CI)** | **p value** |
| --- | --- | --- |
| Male Donor | 0.78 (0.36, 1.67) | 0.5148 |
| Donor Age (per year) | 1.02 (0.99, 1.05) | 0.2011 |
| Deceased Donor | 1.52 (0.73, 3.16) | 0.2609 |
| Pre-emptive Transplant | 0.96 (0.29, 3.18) | 0.9495 |
| Male Recipient | 1.53 (0.72, 3.25) | 0.2662 |
| Recipient Age (per year) | 1.00 (0.98, 1.04) | 0.6892 |
| Recipient Ethnicity (Chinese vs. Other) | 0.84 (0.40, 1.78) | 0.6532 |
| Time from Dialysis to Transplant (per year) | 1.03 (0.95, 1.12) | 0.4483 |
| Delayed Graft Function | 1.40 (0.57, 3.44) | 0.4648 |
| Induction Immunosuppression  (Basiliximab vs. Thymoglobulin) | 1.18 (0.48, 2.92) | 0.7134 |
| Calcineurin Inhibitor (Cyclosporine vs. Tacrolimus) | 2.23 (0.85, 5.85) | 0.1044 |
| Anti-metabolite (Mycophenolate vs. Azathioprine) | 4.60 (0.62, 34.0) | 0.1345 |
| NUCOT Alloimmune Risk Categories |  |  |
| Intermediate vs. Low | 10.4 (1.37, 78.6) | **0.0237** |
| High vs. Intermediate | 4.03 (1.91, 8.52) | **0.0003** |
| High vs. Low | 41.8 (5.43, 322) | **0.0003** |
| *Analysis could not be performed for re-transplants as HLA-DR/DQ dnDSA developed only in recipients receiving their first transplant | | |

**Supplementary Figure 1. Death-censored allograft survival by HLA Class of dnDSA**

Recipients who developed Class II dnDSA with or without Class I had decreased death-censored allograft survival (HR 6.2, 95% CI 1.5-25, p=0.0102) compared to recipients who did not develop dnDSA. No recipients with isolated Class I dnDSA experienced death-censored allograft loss.

**
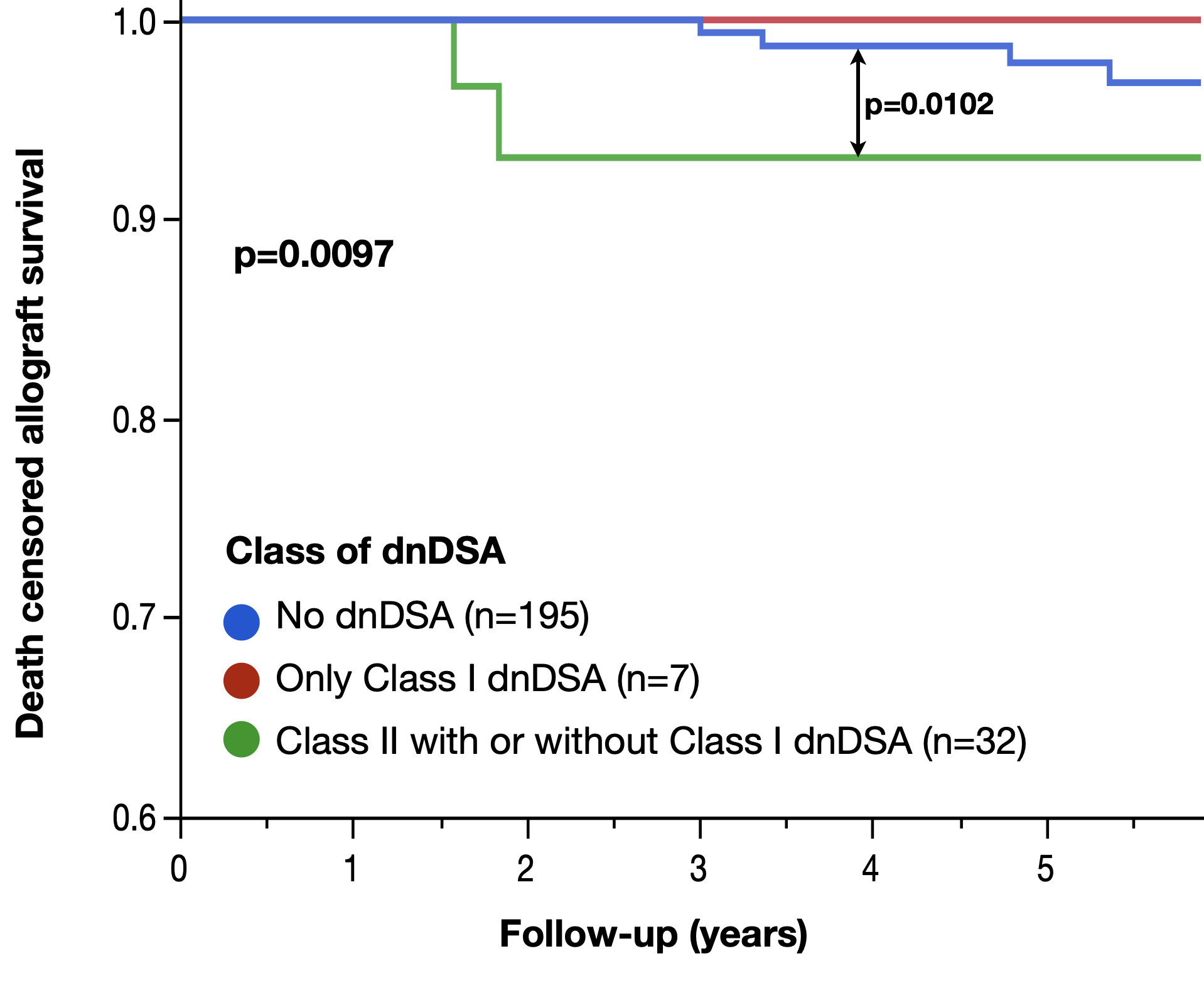
**
